# Impella Versus Intra-aortic Balloon Pump in Cardiogenic Shock: Arrhythmias, Bleeding Complications, and In-Hospital Outcomes in a Propensity-Matched Analysis of the National Inpatient Sample

**DOI:** 10.7759/cureus.114879

**Published:** 2026-08-20

**Authors:** Maha Wazir, Farhan Ullah, Fahad Nasir, Qaidar Alizai

**Affiliations:** 1 Internal Medicine and Nephrology, The Ohio State University Wexner Medical Center, Columbus, USA; 2 Internal Medicine and Nephrology, Ochsner Medical Center, New Orleans, USA; 3 Cardiology, Ochsner Medical Center, New Orleans, USA; 4 Surgery, The Ohio State University Wexner Medical Center, Columbus, USA

**Keywords:** arrhythmias, cardiogenic shock, impella, intra-aortic balloon pump, mechanical circulatory support, national inpatient sample, propensity score matching, ventricular tachycardia

## Abstract

Background: With no robust data indicating a mortality benefit of Impella over the intra-aortic balloon pump (IABP), we conducted a retrospective analysis to assess the frequency of morbidity outcomes, including arrhythmias, associated with the two devices.

Materials and methods: We performed a retrospective observational study using the National Inpatient Sample (NIS) database (2009-2014). Hospitalizations involving acute myocardial infarction or cardiogenic shock, valve surgery, cardiac bypass, or percutaneous coronary intervention, which received Impella or IABP on the day of admission, were identified. Propensity score matching was conducted to account for baseline demographic characteristics and admitting diagnoses. The difference-in-differences statistic was used to assess the difference in arrhythmia outcomes between the two groups. Analyses were performed using RStudio (Posit Software, PBC, Boston, MA, USA). Primary outcomes included arrhythmia rates and in-hospital mortality.

Results: The unmatched data included 5,053 hospitalizations receiving Impella and 133,042 receiving IABP on the day of admission. A 1:1 propensity score matching yielded 10,106 matched hospitalizations. We observed significantly higher odds of ventricular tachycardia (VT) in the Impella group and of supraventricular tachycardia (SVT) in the IABP group. Bleeding, hematoma, and extracorporeal membrane oxygenation (ECMO) use were higher with Impella, whereas iatrogenic cardiac complications and heart transplant were higher with IABP. Subgroup analysis showed higher in-hospital mortality among patients with VF, AF, stroke, and ECMO use in the Impella group.

Conclusions: Our study showed a greater association between VT and Impella, whereas iatrogenic complications and SVT were more commonly associated with IABP use. Impella was associated with significantly higher rates of VT, VF, and in-hospital cardiac arrest and with higher in-hospital mortality, while AF was significantly more common with IABP. These findings suggest a potential benefit of increased post-procedural surveillance in patients receiving Impella, pending confirmation in prospective studies.

## Introduction

Myocardial infarction (MI) complicated by cardiogenic shock remains a life-threatening condition with an extremely high mortality of 40-60% [1,2]. Mechanical circulatory support devices are used short-term to provide hemodynamic support in a variety of circumstances, such as cardiogenic shock and high-risk coronary interventional procedures. These devices support either the left or right side of the heart, depending on the affected chamber. The intra-aortic balloon pump (IABP) is a commonly used device due to its ease of insertion, low cost, and lack of requirement for continuous technical monitoring [2,3]. Impella is another frequently used percutaneous left ventricular assist device (LVAD) that is placed through the femoral artery and via retrograde access across the aortic valve into the left ventricle [4,5].

To date, no studies have demonstrated a morbidity or mortality benefit of Impella over IABP. A recent meta-analysis by Wernly et al., comparing Impella to IABP, did not show any benefit in short-term or 30-day mortality [6]. Still, there is a paucity of large-scale studies comparing the rates of arrhythmias with the two devices and the impact of arrhythmias on mortality in these patients.

Our study aimed to analyze the effect of IABP and Impella on the rates of arrhythmias and mortality in patients admitted with cardiogenic shock. We hypothesized that Impella is associated with higher rates of post-procedural arrhythmias and mortality in these patients. This study aimed to compare rates of post-procedural arrhythmias and in-hospital mortality between patients receiving Impella and those receiving IABP support for cardiogenic shock, hypothesizing that Impella use is associated with higher rates of both outcomes.

## Materials and methods

Data source

We conducted a retrospective observational study using the National Inpatient Sample (NIS) database from 2009 to 2014. This database is provided by the Healthcare Cost and Utilization Project, which collects data from 20% of hospital discharges across all hospitals in the United States using stratified probability sampling. The NIS database provides detailed information for each hospital discharge, including demographics, principal diagnosis, up to 24 secondary diagnoses, and procedural diagnoses.

Inclusion and exclusion criteria

We included all adult patients admitted to the hospital over the study period who were admitted with either acute MI, cardiogenic shock, valve surgery, cardiac bypass, or percutaneous coronary intervention (PCI) and who received Impella or IABP on the day of admission (Day 0). We excluded patients with pre-hospital cardiac arrest.

Patient stratification

Patients were stratified into two groups: Impella and IABP. Propensity score matching was performed to control for demographics, comorbid conditions, vital signs on arrival, and insurance status. The two groups were compared for outcomes post-match.

Outcomes

Our co-primary outcomes included rates of in-hospital arrhythmias (ventricular tachycardia (VT), ventricular fibrillation (VF), atrial fibrillation (AF), and supraventricular tachycardia (SVT)) and in-hospital mortality. Secondary outcomes were length of hospital stay and total hospitalization charges.

Data points

We extracted data about patient demographics (age, gender, and race); comorbid conditions (smoking status, previous cardiac arrest, acute MI, valve surgery, cardiogenic shock, cardiac bypass, previous PCI, alcohol use, congestive heart failure (CHF), chronic pulmonary disease, coagulopathy, diabetes, drug abuse, hypertension, liver disease, neurological disorders, peripheral vascular disease (PVD), pulmonary circulation disorders, and chronic kidney disease); and in-hospital cardiac complications, including cardiac arrhythmias (VT, VF, AF, and SVT); in-hospital cardiac arrest; need for extracorporeal membrane oxygenation (ECMO), LVAD use, and iatrogenic cardiac complications (stroke, gastrointestinal hemorrhage, and peripheral vascular hematomas and bleeding). We also abstracted data on palliative care consults and discharge to hospice care.

Statistical analyses

The primary, secondary, and diagnosis and procedure codes for IABP and Impella insertion were identified using the International Classification of Diseases, Ninth Revision (ICD-9) codes (Table 1). Propensity score matching was performed in a 1:1 ratio, controlling for patient age, race, gender, and comorbidities. Matching was performed using nearest-neighbor matching without replacement, with a caliper width of 0.2 of the standard deviation (SD) of the logit of the propensity score. Post-match, the two groups were compared for demographics, comorbid conditions, and outcomes.

**Table 1:** ICD-9-CM codes used for cohort identification ICD-9-CM: International Classification of Diseases, Ninth Revision, Clinical Modification, IABP: intra-aortic balloon pump, VAD: ventricular assist device, MI: myocardial infarction

Clinical concept	Code type	ICD-9-CM code(s)
Cardiogenic shock	Diagnosis	785.51
Acute MI	Diagnosis	410.xx series
IABP insertion	Procedure	37.61
Impella (percutaneous VAD) insertion	Procedure	37.68

Continuous, normally distributed variables were compared using independent-samples t-tests and reported as means and SDs. Continuous non-normally distributed variables were compared using the Mann-Whitney U test and reported as medians and interquartile ranges (IQR). Categorical variables were compared using the chi-square and Fisher's exact tests. The difference-in-differences statistic was used to assess differences in effect outcomes between the two groups. Further subgroup analysis of outcomes was performed to assess their impact on mortality, length of stay, and hospital charges. For all analyses, p ≤ 0.05 was considered statistically significant, and all analyses were performed using RStudio (Posit Software, PBC, Boston, MA, USA).

## Results

We identified 138,095 hospitalizations, of whom 5,053 (3.7%) received Impella, and 133,042 (96.3%) received IABP on the day of admission (Day 0). After propensity score matching, 10,106 hospitalizations remained (5,053 in each group).

Pre-match baseline characteristics

The mean (SD) age of the study population was 64.8 (10.9) years; 39,177 (28%) were female, and 97,337 (70%) were Caucasian patients. The Impella group had a significantly higher mean age yet a lower proportion of Caucasian and female patients (p < 0.05). Table 2 compares the baseline characteristics of our study groups before and after the match.

**Table 2:** Comparison of baseline characteristics of admitted patients treated with Impella versus patients treated with IABP from 2009 to 2014 The number of participants is based on the sample and is unweighted. Percentages may not total 100 due to rounding and weighting. PCI: percutaneous coronary intervention, MI: myocardial infarction, CHF: congestive heart failure, PVD: peripheral vascular disease, IABP: intra-aortic balloon pump, SD: standard deviation

	Pre-match			Post-match		
	Impella (n = 5,053)	IABP (n = 133,042)	p-value	Impella (n = 5,053)	IABP (n = 5,053)	p-value
Demographics						
Age, mean (SD) years	65.2 (11.2)	64.5 (10.6)	<0.001	65.2 (11.2)	65.7 (11.4)	0.026
Caucasian, n (%)	3,410 (67.5)	93,927 (70.6)	<0.001	3,410 (67.5)	3,395 (67.2)	0.870
Female, n (%)	1,127 (22.31)	38,050 (28.6)	<0.0001	1,127 (22.31)	1,111 (22.0)	0.850
Comorbid conditions, n (%)						
Smoking	909 (18.0)	30,998 (23.3)	<0.001	909 (18.0)	934 (18.5)	0.740
Previous cardiac arrest	91 (1.8)	1,197 (0.9)	<0.001	91 (1.8)	1 (0.02)	0.002
Acute MI	1,455 (28.8)	69,980 (52.6)	<0.001	1,455 (28.8)	1,379 (27.3)	0.100
Valve surgery	0	3 (0.0002)	-	0	0	1.000
Cardiogenic shock	2,081 (41.2)	57,341 (43.1)	0.008	2,081 (41.2)	2,061 (40.8)	0.820
CABG	247 (4.9)	51,886 (39.0)	<0.001	247 (4.9)	349 (6.9)	0.070
History of PCI	3,294 (65.2)	51,088 (38.4)	<0.001	3,294 (65.2)	3,360 (66.5)	0.330
Alcohol use	116 (2.3)	5,055 (3.8)	<0.001	116 (2.3)	116 (2.3)	0.920
CHF	193 (3.82)	3,818 (2.87)	0.005	193 (3.82)	197 (3.9)	0.970
COPD	884 (17.5)	25,011 (18.8)	0.022	884 (17.5)	904 (17.9)	0.790
Coagulopathy	864 (17.1)	24,479 (18.4)	0.015	864 (17.1)	894 (17.7)	0.700
Diabetes, uncomplicated	1,632 (32.3)	39,912 (30.0)	0.005	1,632 (32.3)	1,657 (32.8)	0.820
Diabetes with chronic complications	247 (4.9)	6,386 (4.8)	0.890	247 (4.9)	293 (5.8)	0.390
Drug abuse	131 (2.6)	6,412 (2.0)	0.011	131 (2.6)	131 (2.6)	0.950
Hypertension	3,102 (61.4)	84,880 (63.8)	0.001	3,102 (61.4)	3,153 (62.4)	0.660
Liver disease	96 (1.9)	1,862 (1.4)	0.003	96 (1.9)	76 (1.5)	0.430
Neurological disorders	187 (3.7)	5,321 (4.0)	0.180	187 (3.7)	167 (3.3)	0.700
PVD	757 (15.0)	14,501 (10.9)	<0.001	757 (15.0)	853 (16.9)	0.210
Pulmonary circulation disorders	35 (0.7)	798 (0.6)	0.470	35 (0.7)	30 (0.6)	0.870
Renal failure	1,081 (21.4)	19,158 (14.4)	<0.001	1,081 (21.4)	1,177 (23.3)	0.099

Post-match baseline characteristics

After propensity score matching, the mean (SD) age was 65.4 (11.3) years; 2,238 (22%) were female; and 6,805 (67%) were Caucasian, with no statistically significant difference between the two groups (p > 0.05).

Post-match outcomes

Data on associations between the two groups are summarized in Table 3. The study revealed significantly higher odds of VT (adjusted odds ratio (aOR) = 2.26), hematoma (aOR = 1.62), bleeding (aOR = 2.59), and the need for ECMO (aOR = 6.28) in those who received Impella. Impella was associated with significantly lower odds of SVT (aOR = 0.73) and iatrogenic cardiac complications (aOR = 0.51) than IABP. Impella was associated with significantly higher odds of VF (aOR = 2.02), LVAD use (aOR = 6.62), in-hospital cardiac arrest (aOR = 1.17), and gastrointestinal hemorrhage (aOR = 1.71), and with significantly lower odds of AF (aOR = 0.87) and stroke (aOR = 0.17), compared to IABP (all p < 0.001).

**Table 3:** Multivariable regression analyses for outcomes of Impella versus IABP Values were rounded when doing so did not materially alter the reported ORs or CIs. VT: ventricular tachycardia, IABP: intra-aortic balloon pump, SVT: supraventricular tachycardia, VF: ventricular fibrillation, AF: atrial fibrillation, LVAD: left ventricular assist device, ECMO: extracorporeal membrane oxygenation, OR: odds ratio, CI: confidence interval

Outcome	Adjusted OR (95% CI)	p-value
Any cardiac arrhythmia	1.21 (1.18-1.24)	<0.001
VT	2.26 (2.19-2.33)	<0.001
VF	2.02 (1.94-2.09)	<0.001
AF	0.87 (0.84-0.89)	<0.001
SVT	0.73 (0.70-0.76)	<0.001
Peripheral vascular complications	0.99 (0.89-1.11)	0.908
Hematoma	1.62 (1.51-1.74)	<0.001
Bleeding	2.59 (2.45-2.74)	<0.001
Iatrogenic cardiac complications	0.51 (0.48-0.53)	<0.001
Stroke	0.17 (0.13-0.23)	<0.001
LVAD	6.62 (5.64-7.77)	<0.001
Discharge to hospice care	2.60 (2.45-2.76)	<0.001
Gastrointestinal hemorrhage	1.71 (1.61-1.82)	<0.001
In-hospital cardiac arrest	1.17 (1.10-1.24)	<0.001
ECMO	6.28 (5.63-7.00)	<0.001
Palliative care consult	2.60 (2.45-2.76)	<0.001

Subgroup analyses

Increased incidence of mortality was seen with VF (aOR = 1.54), AF (aOR = 1.39), acute stroke (aOR = 3.14), and in-hospital cardiac arrest (aOR = 2.1) in the Impella cohort. At the same time, ECMO use was associated with lower mortality in the Impella cohort relative to IABP (aOR = 0.35, p = 0.028) (Table 4).

**Table 4:** Subgroup analysis of morbidity and mortality outcomes among hospitalized patients receiving Impella versus IABP (NIS, 2009-2014) Values were rounded when doing so did not materially alter the reported ORs or CIs. VT: ventricular tachycardia, IABP: intra-aortic balloon pump, SVT: supraventricular tachycardia, LVAD: left ventricular assist device, ECMO: extracorporeal membrane oxygenation, PVAD: percutaneous ventricular assist device, LOS: length of stay, OR: odds ratio, VF: ventricular fibrillation, AF: atrial fibrillation, CI: confidence interval, NIS: National Inpatient Sample

Complication	Total number of patients with specific complication in each group	Number of deaths	Total charges	Length of stay		OR for mortality	p-value
				Median	Max		
1. VT							
IABP	693	176	$223677.3	7	305	1.14 (0.91-1.43)	0.242
Impella	996	279	$270106.9	6	52		
2. SVT							
IABP	425	55	$146210.5	6	105	1.49 (0.98-2.26)	0.052
Impella	331	60	$209009.0	7	37		
3. VF							
IABP	383	118	$189721.7	6	154	1.54 (1.146-2.07)	0.003
Impella	451	183	$285049.5	8	48		
4. AF							
IABP	1079	185	$208856.8	9	305	1.39 (1.12-1.74)	0.002
Impella	1048	235	$275244.2	12	69		
5. Any cardiac arrhythmia							
IABP	2007	354	$193093	8	305	1.49 (1.27-1.73)	<0.010
Impella	2084	503	$265213	8	39		
6. In-hospital cardiac arrest							
IABP	200	132	$190050.2	3	94	2.1 (1.28-3.4)	0.002
Impella	179	143	$257298.1	1	26		
7. Acute stroke							
IABP	101	32	$291285	13	119	3.14 (1.89-6.20)	<0.001
Impella	119	74	$571894	10	201		
8. Hematoma							
IABP	164	22	$240080.5	8	85	1.38 (0.79-2.47)	0.247
Impella	333	59	$328674.5	9	135		
9. Hemorrhage							
IABP	110	27	$355463.1	9	127	0.98 (0.57-1.71)	1
Impella	253	63	$389062.3	9	135		
10. ECMO							
IABP	28	20	$533325.9	4	154	0.35 (0.12-0.97)	0.028
Impella	74	34	$548972.4	4	69		

Although the incidence of VT was higher in the Impella group and of SVT in the IABP group, there was no difference in mortality among patients with VT or SVT between the Impella and IABP groups (p > 0.05).

**Figure 1:**
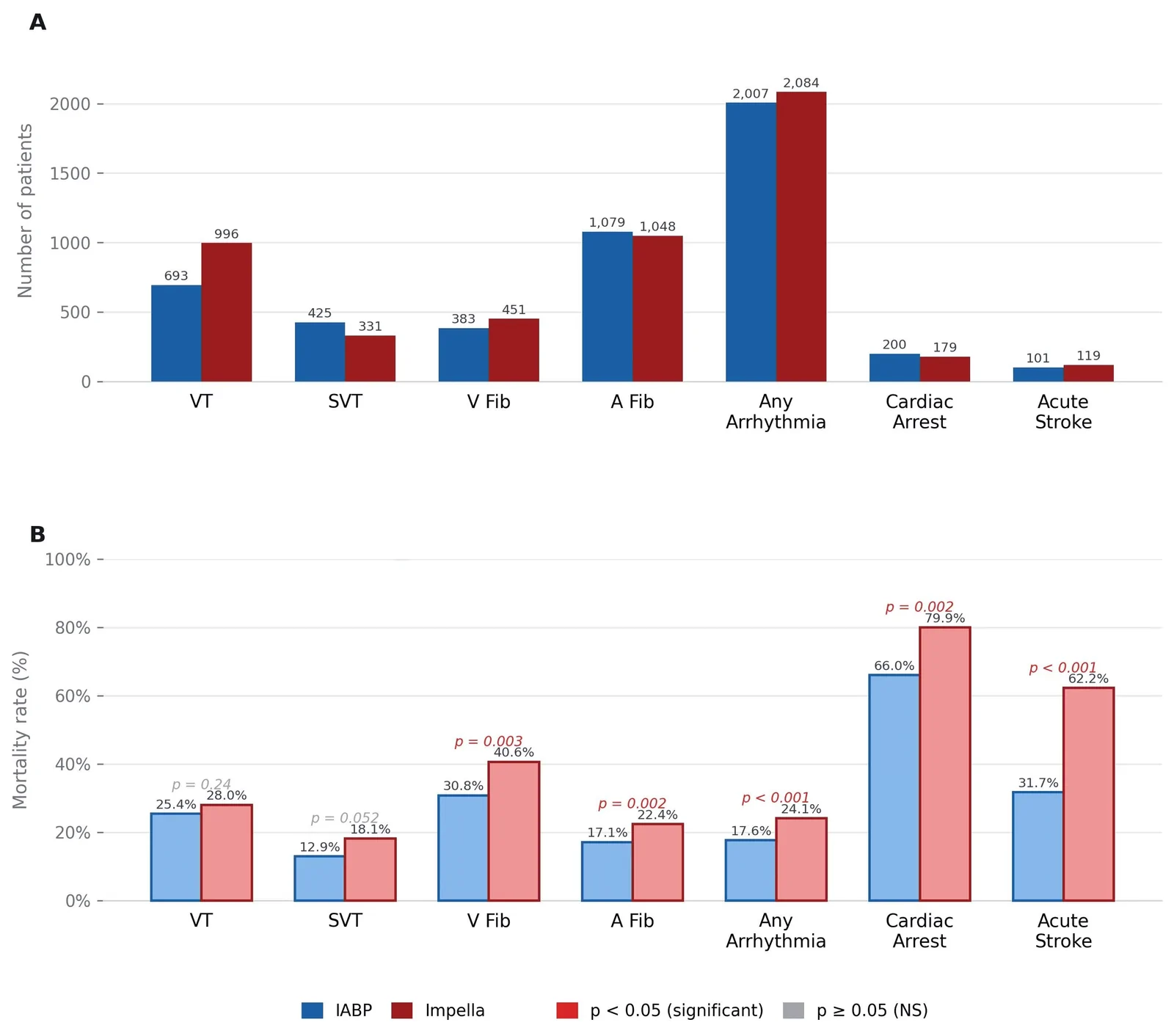
Incidence of arrhythmias and in-hospital mortality comparing IABP versus Impella Panel A shows the number of patients in each device group who experienced the listed complication. Panel B shows in-hospital mortality rates; p-values reflect the difference in mortality between IABP and Impella subgroups for each complication (red = p < 0.05; gray = NS). IABP: intra-aortic balloon pump, VT: ventricular tachycardia, SVT: supraventricular tachycardia, V Fib: ventricular fibrillation, A Fib: atrial fibrillation, NS: not significant

The median length of stay was higher in VT patients on IABP (7 vs. 6 days) and lower in IABP patients with SVT (6 vs. 7 days). A longer length of stay was observed in Impella patients complicated by VF and AF, whereas a similar trend was not observed with IABP (8 vs. 6 days for VF and 12 vs. 9 days for AF). Hospitalization charges were higher across all Impella patient subgroups. Figure 1 depicts the association between various arrhythmias and the two devices and compares mortality between the two subgroups.

## Discussion

IABP has been the most widely used mechanical support device in cardiogenic shock for more than five decades. Registry studies, including those by Scheidt et al., demonstrated improvements in diastolic blood pressure and coronary artery flow, followed by an increase in cardiac output. Similarly, newer devices such as Impella aim to replicate the heart's natural hemodynamics by unloading the left ventricle, thereby decreasing left ventricular end-diastolic pressure and volume [7], which in turn decreases myocardial oxygen demand. Studies have shown improvement in coronary flow velocities and arteriolar resistance with Impella devices. These hemodynamic effects may make Impella useful during selected high-risk coronary interventions. However, recent trials such as the six-year outcome of the IABP-SHOCK II trial demonstrated no difference between IABP and controls in recurrent MI, stroke, repeat revascularization, and hospitalization for cardiac reasons at 30 days or one year [8]. A recent meta-analysis of observational studies showed high 30-day mortality for patients with cardiogenic shock with the use of Impella [9].

Several studies have compared the morbidity and mortality outcomes of Impella versus IABP in hospitalized patients, but few have examined the occurrence of arrhythmias as the primary outcome. Two previous studies, Alushi et al. and O’Neill et al., have reported the incidence of arrhythmias in studies comparing mortality between IABP and Impella [10,11]. While the study by Alushi et al. described the various types of arrhythmias that occurred in each group, the O’Neill et al. (PROTECT II) study used a combined category for the need for cardiopulmonary resuscitation (CPR) or ventricular arrhythmias. However, neither study showed any statistically significant difference in arrhythmias between the two populations. In the PROTECT II study, patients who underwent CPR >20 min before device insertion had worse 30-day mortality (p = 0.001). Based on this finding, we chose to exclude patients with out-of-hospital cardiac arrest.

Our study demonstrated an increased incidence of VT in the Impella subset, with no significant difference in mortality between the two groups. The higher observed incidence of VT with Impella versus IABP might be related to mechanical irritation of adjacent cardiac structures by the device [12,13]. These complications may be avoided with echocardiographic surveillance during and after Impella placement. Precise placement and positioning of the device are prerequisites for successful functioning [13]. The inlet should be 3.5 cm distal to the aortic valve with a positioning marker at the valve, an Impella catheter along the inner curve of the aorta, and the wire in the left ventricular apex. Any misplaced device in the left ventricle can precipitate potentially life-threatening arrhythmias; this risk might be mitigated by removing excess slack from the catheter in the ascending aorta or arch of the aorta [13].

The incidence of SVT was lower in the Impella group, but there was no statistically significant association with mortality compared with IABP. In contrast, our study showed that Impella was associated with significantly higher odds of VF and in-hospital cardiac arrest and significantly lower odds of AF and acute stroke relative to IABP; mortality was also higher in the Impella cohort within each of these subgroups. Whether the increased mortality is directly related to the device or whether it could be due to a higher use of Impella in sicker patients is unknown. Contrary to the expectation that Impella is used in sicker patients, data from the Premier Healthcare Database showed a lower use of Impella in critically ill patients [14].

Our study also demonstrated a significantly lower risk of SVT and fewer iatrogenic cardiac complications in the Impella group. Studies from the Europella Registry showed that Impella was associated with a decreased risk of iatrogenic complications compared with older devices [15]. As high-risk PCIs can result in hemodynamic compromise or electromechanical dissociation, device selection is critical, as it can dramatically reduce PCI-associated ischemic time. Previous observational data from three small case series on the use of Impella 2.5 demonstrated good peri-procedural safety and operability [7,16,17]. Although large-scale studies on such peri-procedural comparisons are lacking, these studies suggest that successful percutaneous coronary revascularization with peri-procedural Impella implantation may not have long-term adverse effects.

Similarly, regarding the risk of increased perioperative bleeding in the Impella subgroup, our findings are in line with previous studies [18]. The higher observed bleeding rate in the Impella group may be attributed to the use of larger delivery system sizes (i.e., 12- and 13-French sheaths vs. 7-8-French sheaths used with IABP). It may be mitigated by using a smaller delivery system [6]. Another study by Boudolas et al., however, revealed no significant differences in bleeding between the two groups, with the major source of bleeding identified as peri-procedural. However, 30.7% of these patients also had bleeding from gastrointestinal sources; this finding may warrant the use of H2 blockers or proton pump inhibitors in such patients [19]. Similarly, the use of Impella as an adjunct to veno-arterial ECMO (VA-ECMO) has been described in various studies, with results demonstrating better hemodynamic outcomes than VA-ECMO alone [5,20]. Boudoulas et al. also demonstrated no significant differences in mortality between the two groups [19]. Data from several other trials revealed improved survival with early implantation of these devices (before PCI), with a significant benefit at door-to-Impella times ≤48 minutes [21,22].

Although hematoma and bleeding complications were significantly higher in the Impella group, interestingly, no statistically significant difference was found in peripheral vascular complications between the two cohorts. In contrast to our study, Schrage et al. demonstrated a higher rate of peripheral vascular complications in the Impella group (10% vs. 4%; p = 0.01) [23]. This was attributed to the size of the delivery system, as in the case of bleeding. Besides using a smaller delivery system, using the Impella 5.0 might improve outcomes by enhancing peripheral perfusion with a higher flow rate of 5 L/min. This requires surgical intervention, which, compared with percutaneous Impella 2.5 implantation, requires more time and expertise [6].

Limitations

This study has several limitations. Because device selection was not randomized, our findings should be interpreted as associations rather than causal effects. The study population was also heterogeneous, pooling patients admitted with five distinct primary diagnoses (acute MI, cardiogenic shock, valve surgery, cardiac bypass, and PCI), which likely carry differing risk profiles that propensity matching on baseline covariates cannot fully reconcile. Disease severity and left ventricular ejection fraction were not captured in the NIS and could not be included in matching or adjustment; patients selected for Impella may have differed systematically in illness severity in ways our model could not capture, so the observed VT and mortality associations could partly reflect unmeasured severity rather than a device effect.

Outcome and comorbidity ascertainment relied on ICD-9-CM administrative coding, which is subject to misclassification and to variation in coding practice across hospitals and over time. We also cannot confirm whether arrhythmias occurred before or after device placement; we mitigated this by restricting inclusion to patients who received their device on the day of admission, but residual timing uncertainty remains, and rhythm-outcome associations should be interpreted cautiously. Only in-hospital mortality and outcomes were available, with no long-term follow-up.

Taken together, these constraints mean our findings should be viewed as hypothesis-generating rather than definitive evidence of a causal device effect, pending confirmation in prospective studies or analyses with more complete risk adjustment. Despite these limitations, this study offers one of the larger national-database analyses to specifically examine arrhythmia outcomes with Impella and IABP use and their relationship to mortality.

## Conclusions

Our study found notable differences in arrhythmia incidence and complications between patients receiving Impella versus IABP support. Impella placement was associated with higher VT and lower SVT rates, though mortality was similar between the groups for these arrhythmias. Additionally, bleeding events occurred more frequently with Impella. In contrast, IABP patients had significantly higher rates of iatrogenic cardiac complications, while ECMO use was significantly more common in the Impella group.

Impella may offer hemodynamic advantages over IABP in certain settings; however, its association with significantly higher rates of ECMO use and bleeding underscores the need for careful patient selection, access planning, and vigilant post-procedural monitoring. IABP may pose a lower risk of arrhythmia, though confirmation from large randomized trials is needed.

Overall, our real-world data indicate no clear superiority of one device over the other. Individualized circulatory support plans that factor in arrhythmia history, bleeding risk profiles, and institutional expertise appear prudent, rather than a universal recommendation for a single device. Larger comparative effectiveness analyses would better inform guidelines for tailored device selection in patients with cardiogenic shock. With the increased availability of percutaneous assist options, defining device-specific risk-benefit profiles is important for optimizing patient selection and clinical outcomes.
